# Clinical Implications of Point-of-Care Measurement of Adalimumab Concentration and Anti-Adalimumab Antibodies in Patients with Rheumatoid Arthritis and Ankylosing Spondylitis

**DOI:** 10.3390/ijms26178741

**Published:** 2025-09-08

**Authors:** Jiyeol Yoon, Jason Jungsik Song, Sang-Won Lee, Hee Jin Park, Yong-Beom Park

**Affiliations:** 1 Division of Rheumatology, Department of Internal Medicine, Yonsei University College of Medicine, Seoul 03722, Republic of Korea; jiyoonmd@yuhs.ac (J.Y.); jsksong@yuhs.ac (J.J.S.); sangwonlee@yuhs.ac (S.-W.L.); phj0111@yuhs.ac (H.J.P.); 2 Institute for Immunology and Immunological Diseases, Yonsei University College of Medicine, Seoul 03722, Republic of Korea; 3 Brain Korea 21 Plus Project for Medical Science, Yonsei University College of Medicine, Seoul 03722, Republic of Korea

**Keywords:** adalimumab, anti-adalimumab antibody, rheumatoid arthritis, ankylosing spondylitis, therapeutic drug monitoring, point-of-care

## Abstract

To evaluate the analytical performance and clinical utility of the automated fluorescence-based POC immunoassay system (AFIAS), compared with established enzyme-linked immunosorbent assay (ELISA) methods for measuring adalimumab and anti-adalimumab antibodies (AAAs) in patients with rheumatoid arthritis and ankylosing spondylitis. 96 patients receiving adalimumab for rheumatoid arthritis (RA) or ankylosing spondylitis (AS) were consecutively recruited. Measurements of adalimumab trough levels and AAAs were taken before the patients’ scheduled adalimumab injection. Three ELISA techniques (RIDASCREEN^®^, IDKmonitor^®^, and LISA TRACKER) were compared with the AFIAS method. Statistical analyses included Bland–Altman, Passing–Bablok regression, kappa values, and intraclass correlation coefficients. Clinical and demographic characteristics were examined to determine the association between adalimumab concentration and AAA detection. The diagnoses included 58 RA diagnoses and 38 AS diagnoses. The median concentrations were 9.33, 7.4, 7.4, and 9.38 µg/mL for RIDASCREEN, IDKmonitor, LISA TRACKER, and AFIAS, respectively. Strong correlations were observed between the techniques. Bland–Altman analysis revealed bias differences of 0.85, 2.03, and 2.76 µg/mL, and the Passing–Bablok regression slopes were 1.046, 1.391, and 1.274 for RIDASCREEN, IDKmonitor, and LISA TRACKER, respectively, compared with AFIAS. Agreement in AAA detection showed kappa values of 0.81 and 0.75 for AFIAS versus IDKmonitor and LISA TRACKER, respectively. A high body mass index, extended injection interval, and RA diagnosis were associated with low adalimumab concentrations in the multivariate analysis. Antinuclear antibody positivity, a higher rheumatoid factor, and disease activity were associated with AAA positivity in univariate analysis. The AFIAS POC measurement method demonstrated time-efficient and highly agreeable results for adalimumab and AAA measurements compared with the results of commercial ELISA methods.

## 1. Introduction

Anti-tumor necrosis factor (TNF) inhibitors have revolutionized the management and prognosis of various inflammatory diseases [1]. However, some patients initially resist treatment, or experience a decline in efficacy over time. For example, a previous study reported the failure of adalimumab, a monoclonal human antibody, to maintain adequate drug concentrations in secondary non-responders, with the potential formation of antibodies against the drug [2]. Consequently, assessing drug concentrations and detecting anti-drug antibodies in patients with suboptimal drug responses may offer a more precise understanding of their condition and guide subsequent treatment strategies [3]. The measurement of drug concentrations and antibodies is predominantly conducted using enzyme-linked immunosorbent assay (ELISA) methods, which, despite their high validity, are constrained by the necessity of pooling sera from multiple patients, the requirement for kit calibration, and the extended time needed to obtain results, often rendering them impractical for same-day clinical decision-making [4]. Given these shortcomings of ELISA, point-of-care (POC) medical technologies are gaining popularity because of the increasing trend toward individualized medical services, where the prompt application of assessment results enhances time and cost efficiency. The AFIAS platform represents a promising POC solution, utilizing automated fluorescence-based detection with cartridge-based assays for both drug concentration and anti-drug antibody measurements.

This study aimed to assess the analytical performance and clinical utility of the AFIAS automated fluorescence-based point-of-care (POC) immunoassay system, employing the AFIAS-10 device with adalimumab and anti-adalimumab antibody cartridges, compared with established ELISA methods. Clinical factors associated with low drug concentrations and antibody emergence were also evaluated.

## 2. Results

### 2.1. Precision and Accuracy

The analytical performance of the AFIAS Adalimumab assay (Boditech Med Inc., Chuncheon, Republic of Korea) was evaluated according to the CLSI (Clinical & Laboratory Standard Institute) guidelines. The precision of the AFIAS assays for adalimumab was assessed across four levels of expected concentrations (2, 5, 8, and 20 µg/mL). The results showed repeatability, within-laboratory precision, and lot-to-lot precision of ≤7.8%, ≤7.1%, and ≤6.6%, respectively (Table 1). The accuracy of the AFIAS adalimumab assay was estimated using the World Health Organization international standard material (NIBSC code 17/236), which was diluted to six different levels and tested using three different lots of cartridges. The recovery rates of the expected values were between 94% and 97% (Table 2).

Additionally, inter-operator variability was evaluated by having two trained laboratory technicians independently perform all AFIAS measurements under standardized protocols. Inter-operator precision showed high reproducibility with coefficients of variation ≤9.2% across four concentration levels (Supplementary Table S1).

### 2.2. Demographics

This study included 96 patients, comprising 42 males and 54 females, with a median age of 53.5 years. A total of 58 patients had serum rheumatoid arthritis (RA), while 38 patients had ankylosing spondylitis (AS). Among these patients, 67 received injections of the original Humira^®^ (AbbVie Inc., North Chicago, IL, USA), whereas 29 received injections of the generic adalimumab product Adalloce^®^ (Samsung Bioepis, Incheon, Republic of Korea). The mean administration duration was 5.0 ± 4.4 years, with the majority (91%) of patients receiving injections biweekly (Table 3). When divided into two groups, the RA group was characterized by older age; more women; positive antinuclear antibody (ANA) response; rheumatoid factor (RF); anti-cyclic citrullinated peptide antibodies; a higher erythrocyte sedimentation rate (ESR); and a combination of methotrexate and corticosteroid. In contrast, the AS group had a longer adalimumab injection interval (Table 3).

### 2.3. Drug Concentration

**Figure 1:**
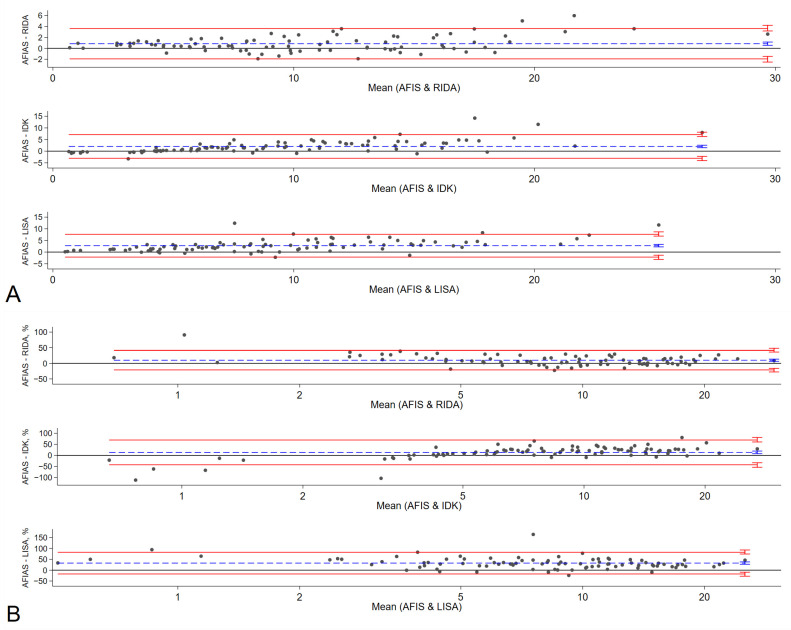
Bland–Altman Chart. Bland–Altman analysis verified the difference in ADL measurements among RIDA, IDK, LISA, and AFIAS (**A**). Percentage differences between AFIAS and RIDA, IDK, and LISA are shown in (**B**). The difference between the two measurements (**A**) and the percentage difference (**B**) are plotted on the *y*-axis, and the average of the two measurements is plotted on the *x*-axis. Blue lines represent the bias, and red lines represent the 95% limit of agreement (LOA) for each comparison. Small vertical bars at the end of each line represent 95% confidence intervals. AFIAS—AFIAS Adalimumab; IDK—IDKmonitor^®^ Adalimumab Drug Level; LISA—LISA-TRACKER Duo Adalimumab; RIDA—RIDASCREEN^®^ ADM monitoring.

**Figure 2:**
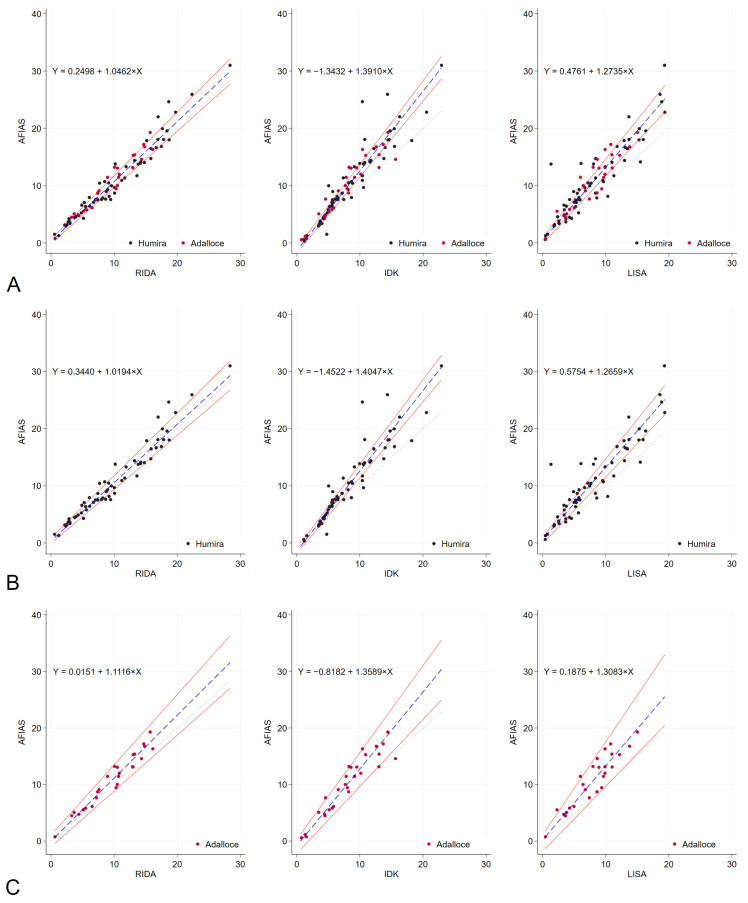
Passing–Bablok regression analysis results. Passing–Bablok regression analysis was conducted to compare the performance of RIDA, IDK, LISA, and AFIAS with regard to the measurement of adalimumab levels across all patients (**A**). Additionally, the analysis was stratified based on the type of adalimumab administered, either the original Humira^®^ (**B**) or the generic Adalloce^®^ (**C**). The dotted lines represent the reference line (y = x), whereas the solid red lines indicate 95% confidence bounds. The blue dashed lines illustrate the Passing–Bablok regression lines. The regression formula is shown in the upper left corner of each plot. (**A**): For all patients, (**B**): original adalimumab (Humira^®^) and (**C**): generic adalimumab (Adalloce^®^). AFIAS—AFIAS Adalimumab; IDK—IDKmonitor^®^ Adalimumab Drug Level; LISA—LISA-TRACKER Duo Adalimumab; RIDA—RIDASCREEN^®^ ADM Monitoring.

The results obtained from the POC method, specifically the AFIAS Adalimumab assay, were compared with those of three established commercial ELISA methods: RIDASCREEN^®^ ADM Monitoring (R-Biopharm AG, Darmstadt, Germany), IDKmonitor^®^ Adalimumab Drug Level (Immundiagnostik AG, Bensheim, Germany), and LISA-TRACKER Duo Adalimumab (Theradiag, Croissy-Beaubourg, France). The median adalimumab concentrations were 9.38 μg/mL with AFIAS Adalimumab assay and 9.33, 7.4, and 7.4 μg/mL with RIDASCREEN^®^ ADM Monitoring, IDKmonitor^®^ Adalimumab Drug Level, and LISA-TRACKER Duo Adalimumab, respectively (Supplementary Table S2). Compared to AFIAS, the corresponding Pearson correlation coefficients for RIDASCREEN, IDKmonitor, and LISA were 0.9736, 0.9336, and 0.9233. Bland–Altman charts showed that AFIAS had a tendency to overestimate adalimumab concentration compared to established ELISA methods; the mean differences between AFIAS and RIDASCREEN, IDKmonitor, and LISA were 0.85, 2.03, and 2.76 μg/mL, respectively (Table 4, Figure 1). The differences and percentage differences were acceptable when the pairs were within the therapeutic adalimumab concentrations, which were defined as 5–12 μg/mL. The Passing–Bablok regression (Figure 2, Table 4) showed baseline bias for IDKmonitor (constant A, −1.3432, 95% confidence interval [CI] −1.7357 to −0.868), and proportional bias (slope 1.391, 95% CI 1.324 to 1.458). For LISA, proportional bias (slope, 1.2735, 95%CI 1.1893 to 1.3704) existed compared to AFIAS. No significant difference was observed between the original (Humira^®^) and generic adalimumab (Adalloce^®^) in correlation with established ELISA methods. Still, AFIAS showed that generic adalimumab had a more overestimated value than the original adalimumab when compared to the value obtained through ELISA (slope 1.1116 vs. 1.0194 for RIDA, 1.3083 vs. 1.2659 for LISA). This was compared to AFIAS, Lin’s concordance correlation coefficients of absolute agreement, which were 0.9595, 0.8291, and 0.7939 for RIDASCREEN, IDKmonitor, and LISA, respectively. Further, compared with AFIAS, the ICCs were 0.97, 0.89, 0.90, and 0.90 for RIDASCREEN, IDKmonitor, LISA, and their combination, respectively, suggesting that RIDASCREEN revealed the most similar results for measuring the adalimumab concentration compared to AFIAS (Table 5). When divided by the arbitrary level of a recommended therapeutic concentration of 5–12 μg/mL, with <5 μg/mL as subtherapeutic, 5–12 μg/mL as therapeutic, and >12 μg/mL as supratherapeutic, RIDASCREEN had the best kappa value of 0.857 (overall agreement 91%, Supplementary Table S3). Likewise, when the measurement pairs were plotted by frequency and difference, the AFIAS method for adalimumab measurement was closest to RIDASCREEN, but somewhat distant from LISA (Supplementary Figure S1).

### 2.4. Detection of Anti-Drug Antibodies

The detection of AAA using two enzyme-linked immunosorbent assay (ELISA) methods—IDKmonitor^®^ Adalimumab free ADA (Immundiagnostik AG, Darmstadt, Germany) and LISA-TRACKER Duo Adalimumab (Theradiag, France)—was compared to the POC method, AFIAS Free Anti-Adalimumab (Boditech, Chuncheon, Republic of Korea). AAAs were detected in 13, 12, and 11 patients using IDKmonitor^®^ Adalimumab free ADA, LISA-TRACKER Duo Adalimumab, and AFIAS Free Anti-Adalimumab, respectively. Sixteen patients showed positive results across any of the three methods (IDKmonitor, LISA, AFIAS), whereas nine patients showed positive results for all three methods. The overall, positive, and negative percent agreement of AAA detection with AFIAS for IDKmonitor were 96%, 77%, and 99%, respectively, and those for LISA were 95%, 75%, and 98%, respectively.

Cohen’s kappa values were 0.81 for AFIAS vs. IDKmonitor, 0.75 for AFIAS vs. LISA, and 0.77 for LISA vs. IDKmonitor. Overall, the results demonstrated good agreement, with a Fleiss kappa value of 0.78 (Table 5). Regarding AAA detection, IDKmonitor showed almost perfect agreement with the AFIAS.

### 2.5. Clinical Correlation and Implications

The rheumatoid and non-rheumatoid arthritis groups showed no difference in adalimumab concentration, but AAA positivity tended to be higher in the rheumatoid arthritis group, especially when measured by IDKmonitor or LISA (Supplementary Table S3).

**Figure 3:**
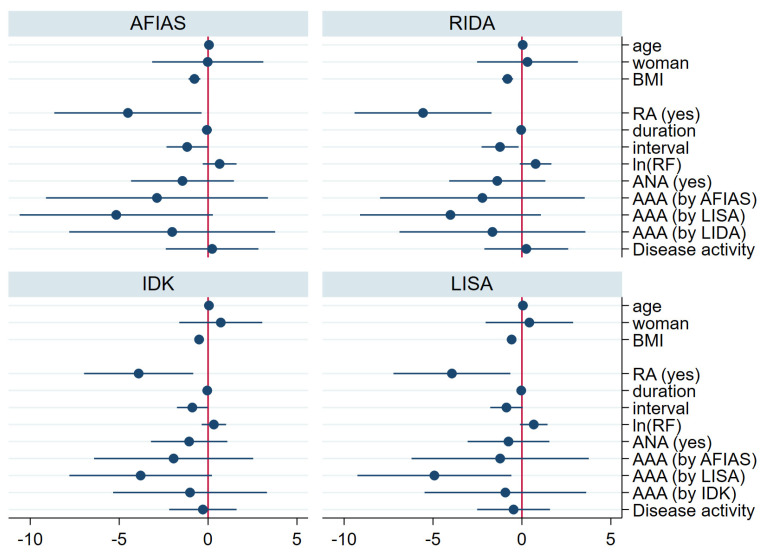
Regression coefficient for drug concentration by each kit (multivariate analysis). In the multivariate regression analysis for the drug concentration, higher BMI, prolonged injection interval, and diagnosis of RA were associated with low trough drug concentration. AAA—anti-drug antibody; ANA—antinuclear antibody; BMI—body mass index; duration—years from the first adalimumab injection to the day of enrollment; interval—interval between adalimumab injections; ln(RF)—log natural function of quantification of rheumatoid factor; RA—rheumatoid arthritis; AFIAS—AFIAS Adalimumab or AFIAS Free Anti-Adalimumab; IDK—IDKmonitor^®^ Adalimumab Drug Level or IDKmonitor^®^ Adalimumab Free ADA; LISA—LISA-TRACKER Duo Adalimumab; RIDA—RIDASCREEN^®^ ADM Monitoring.

Regarding adalimumab concentrations, univariate regression analysis revealed significant negative correlations with a higher body mass index (BMI), longer intervals between adalimumab injections, and AAA positivity. Furthermore, multivariate regression analysis indicated that a diagnosis of RA, a higher BMI, and a longer injection interval were significantly negatively correlated with drug concentration (Figure 3, Supplementary Figure S2).

**Figure 4:**
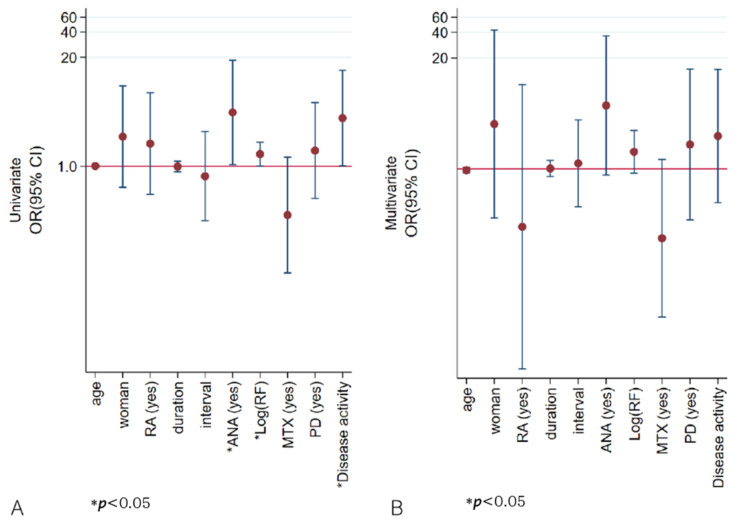
Odds ratios for the association between anti-drug antibody detected via AFIAS and selected variables. In univariate logistic analysis (**A**), ANA, higher RF values, and higher disease activity were associated with AAA positivity, but in multivariate analysis (**B**) none was significant. The *Y*-axis is plotted on a logarithmic scale. ANA—antinuclear antibody; duration—years from the first adalimumab injection to the day of enrollment; interval—interval (weeks) between adalimumab injections; ln(RF)—log natural function of rheumatoid factor quantification; MTX—methotrexate; PD—prednisolone; RA—rheumatoid arthritis; AFIAS—AFIAS Free Anti-Adalimumab.

Patients with AAA positivity were more likely to have ANAs and increased disease activity, showing a trend for increased ESR and higher RF. In the univariate analysis, ANA, RF, and disease activity were associated with AAA positivity, while multivariate analysis showed no significant factors (Table 6, Figure 4, Supplementary Table S5). In the application of backward elimination within multivariate logistic regression, ANA positivity remained a significant predictor of AAA positivity, with an odds ratio of 5.26 and a *p*-value of 0.039. Furthermore, female sex, a diagnosis of rheumatoid arthritis, higher RF value, ANA or AAA positivity, and a combined treatment involving prednisolone were related to disease activity in the univariate logistic analysis. However, in the multivariate analysis, no significant association was observed. (Supplementary Figure S3).

### 2.6. Longitudinal Follow-Up Results

**Figure 5:**
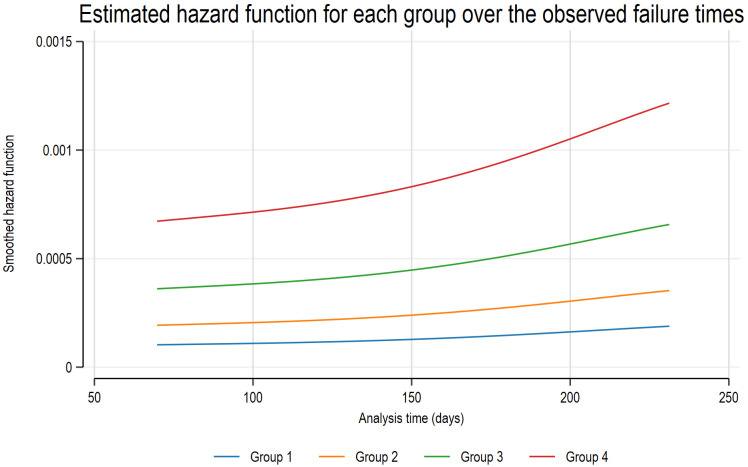
Estimated hazard function for each group over the observed failure times. Patients were categorized based on an ADA concentration threshold of 5 µg/mL and the presence of AAA into four groups: group 1 (high ADA, AAA−), group 2 (high ADA, AAA+), group 3 (low ADA, AAA−), and group 4 (low ADA, AAA+). Notably, patients in group 4, characterized by low ADA concentrations and positive AAA, were most frequently associated with ADA discontinuation during follow-up. AAA—anti-adalimumab antibody; ADA—adalimumab.

The patients were monitored from the date of enrollment until they transitioned from adalimumab to alternative biologics or targeted synthetic DMARDs. Over the course of 280 ± 64 days of follow-up, 8 out of 96 patients transitioned to alternative b/tsDMARDs due to the ineffectiveness of adalimumab during this period (Supplementary Table S6). Among these, six patients transitioned to a different type of b/tsDMARDs, while two patients switched to other TNF inhibitors. When categorizing the patients into four groups (group 1: high ADA concentration and negative AAA; group 2: high ADA concentration and positive AAA, group 3: low ADA concentration and negative AAA; group 4: low ADA concentration and positive AAA, using the threshold of 5 µg/mL as specified in Section 2.4), a clinically relevant association with ADA discontinuation was observed during the follow-up (Figure 5 and Supplementary Figure S4). In a multivariate Cox regression analysis, older age and shorter duration of ADA treatment emerged as risk factors for ADA discontinuation (Supplementary Table S7).

## 3. Discussion

In this study, the newly developed lateral-flow POC cartridge for measuring serum adalimumab concentration and anti-drug antibodies demonstrated clinically applicable levels of accuracy and reliability compared with those of existing commercially available ELISA devices. The difference in adalimumab concentration was acceptable for clinical utility based on the Bland–Altman plot of difference, as the outlier was plotted at a higher mean concentration than the usual therapeutic cut-off value of 5–12 μg/mL [5,6,7]. The detection of AAAs was largely consistent across the three kits, although some patients exhibited varying results between kits, a phenomenon that was confirmed by the commercially available kits. The POC cartridge also exhibited consistent characteristics with respect to patient clinical profiles, rendering it suitable for clinical application. The presence of ANAs correlates with patient immunological characteristics, is more prevalent in women, and is associated with autoimmune diseases [8]. In this study, positive ANA results were pre-tested in all patients and were not related to the use of TNF inhibitors. In univariate analysis, ANA was found to be associated with AAA development. However, this association was not evident in multivariate analysis, with a small margin of difference. This discrepancy may be attributed to the limited sample size of the patient cohort. These findings indicate that baseline ANA positivity is associated with AAA development, which is consistent with the findings of previous research. Indeed, previous studies have shown that patients with baseline ANAs and higher titers prior to commencing TNF inhibitors were more likely to develop anti-drug antibodies [8]. In addition, lower drug concentrations were observed in patients with longer intervals between injections or higher BMI [2,9]. This expands beyond prior work that evaluated the AFIAS platform with one infliximab and one adalimumab ELISA in patients with various rheumatic diseases [10]. Our comprehensive analytical validation employs multiple agreement metrics, providing deeper insight into analytical performance. Furthermore, we extend the clinical utility evaluation by examining patient-specific factors associated with adalimumab trough levels and anti-adalimumab antibody positivity through multivariate analyses, addressing knowledge gaps.

Current methods measure the levels of free anti-drug antibodies but do not detect the bound form of anti-drug antibodies. This implies that certain antibodies may not be detected because they bind to the drug, even though the patient has developed AAA. Notably, this phenomenon is likely to occur in environments with high drug concentrations. This explains why AAA is rarely observed in patients with elevated drug levels, yet it is detected in those with lower concentrations with current method.

An important limitation of the AFIAS method is the detection of only free anti-adalimumab antibodies, whereas drug-tolerant assays can detect both free and bound AAA. This limitation has significant clinical implications for therapeutic decision-making. Recent studies have demonstrated that drug-tolerant assays detect AAA in 63–90% of patients compared to 21–30% using drug-sensitive methods such as AFIAS [11,12,13]. In patients with high circulating adalimumab levels, free AAA may be undetectable despite the presence of clinically relevant bound antibodies that form immune complexes. These immune complexes can lead to increased drug clearance and predict future treatment failure, even when trough drug levels appear therapeutic [2]. The clinical consequence of this limitation is the potential for false-negative AAA results in patients who may benefit from treatment modification. Patients with bound AAA detected by drug-tolerant assays show increased risk of treatment failure within 6–12 months, even when free AAA assays remain negative [11,14]. This suggests that AFIAS results should be interpreted with caution, particularly in patients with adequate drug levels but suboptimal clinical response. For optimal clinical decision-making, the combination of free AAA measurement (as provided by AFIAS) with drug concentration assessment may help identify at-risk patients. However, clinicians should be aware that negative free AAA results do not exclude the presence of clinically relevant immunogenicity, particularly in patients with sustained high drug levels or those being considered for dose reduction.

In general, the detection of anti-drug antibodies can be performed using various assay methodologies, including radioimmunoassays, ELISA, and cell-based reporter gene assays, each with distinct characteristics. The choice of assay significantly influences the types of antibodies detected and their clinical interpretation [4,15,16]. Among them, specially designed drug tolerance assays can detect both free and binding forms of AAAs but require an additional step to isolate binding anti-drug antibodies, which increases the cost of testing [12,16,17]. However, the additional benefits and utility of measuring total anti-drug antibodies compared to free antibodies remain unclear [18]. Furthermore, no standardized and validated method is currently available to distinguish between more potent and weak neutralizing antibodies, regardless of the binding or free form of AAAs [13,19,20]. Therefore, the measurement of both free and binding AAAs does not necessarily explain all cases in which treatment remains effective or has reduced effectiveness [21]. However, in patients with reduced drug effectiveness, detection of reduced drug concentrations and anti-drug antibodies may guide the modification of treatment strategies. For instance, high drug concentrations and the absence of anti-drug antibodies, coupled with high disease activity, suggest an inflammatory response pathophysiology in which TNF inhibitors have little efficacy, warranting consideration of switching to biologics with a different mechanism or synthetic-targeted disease-modifying anti-rheumatic drugs [4]. Conversely, if the drug concentration is low and the antibodies are not detected, adjusting the dose or dosing interval may be appropriate. In some cases, remission can be achieved with a low drug concentration, absence of anti-drug antibodies, and low disease activity [22]. However, it remains unclear whether the trough adalimumab concentration and AAA detection warrant the modification or switching of therapeutic modalities to maintain achievable disease control. Some reports have shown that trough drug concentration and detection of anti-drug antibodies have poor predictability of disease activity in the real world [3,23].

This analysis revealed some differences between the measuring devices, but these differences were within clinically acceptable ranges and were not specifically attributable to lower POC device specificity, as the differences were also observed among commercially available kits. However, the observed discrepancy, particularly at supratherapeutic concentration levels, which was measured to be larger with POC devices, should be taken into account before deciding to reduce the dosage or extend the injection interval.

The clinical implementation of point-of-care (POC) therapeutic drug monitoring represents a paradigm shift from traditional laboratory-based approaches in rheumatology practice. Recent evidence from inflammatory bowel disease demonstrates that proactive TDM significantly improves clinical outcomes and enables efficient drug de-escalation in patients achieving remission [24]. The AFIAS platform’s 10–12 min turnaround time addresses a critical workflow limitation, as conventional ELISA-based TDM requires 1–3 weeks for results, often necessitating multiple patient visits and delayed treatment adjustments [25,26]. This rapid availability enables same-day clinical decision-making, potentially reducing follow-up visits, treatment delays, and overall healthcare costs, though specific cost-reduction percentages require validation in rheumatology populations. The integration of POC TDM into outpatient settings requires consideration of staff training, quality management systems, and regulatory compliance, but offers the potential to transform reactive, empirically guided treatment approaches into proactive, data-driven therapeutic optimization [27]. Future integration with electronic health records and clinical decision support systems may further enhance the clinical utility of POC TDM platforms, supporting the development of personalized biologic therapy algorithms and advancing precision medicine in rheumatology practice.

**Figure 6:**
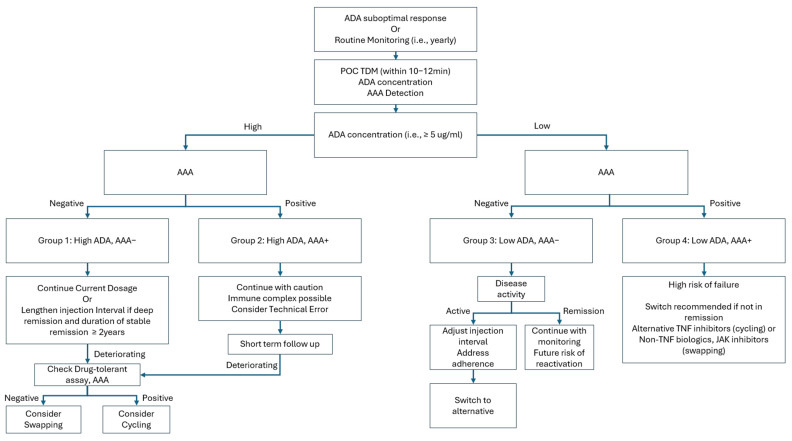
Flow diagram implementation of point-of-care measurement of ADA and AAA. AAA—anti-adalimumab antibody; ADA—adalimumab; POC—point of care; TDM—therapeutic drug monitoring; TNF—tumor necrosis factor; JAK—Janus kinase; Cycling—switching to other TNF inhibitors other than adalimumab; Swapping—switching to the other biologics or JAK other than TNF inhibitors.

In the context of real-world clinical practice, the measurement of drug concentration or the detection of anti-drug antibodies should not be interpreted as a single binary decision-making tool. As demonstrated by the longitudinal drug retention results in this study, a low drug concentration and the presence of anti-drug antibodies are indeed associated with an increased likelihood of drug discontinuation and the transition to alternative agents. However, this process does not occur within a single time frame; rather, it exhibits a gradual trajectory, indicating a higher probability of eventual drug retention failure. In cases where a patient is in deep remission, the immediate likelihood of reactivation is minimal, even in the presence of low drug concentrations and anti-drug antibodies. However, the potential for future reactivation remains. This underscores the importance of evaluating cost-effective strategies to identify individuals who are most likely to benefit from these assessments. We illustrate this implication in Figure 6.

This study has some limitations. First, the study had a relatively small sample size. Although more exceptional cases might emerge in a larger cohort, it remains challenging to ascertain whether the observed bias is unique to the patients in this study. Second, the clinical significance of measuring the total anti-drug antibodies remains unclear. This method measures free anti-drug antibodies; however, detecting these antibodies becomes challenging as the drug concentration increases compared to the total anti-drug antibody measurement. However, the measurement of total anti-drug-antibody levels does not confirm the specific functional aspect of the neutralizing degree, further complicating its clinical relevance. Third, the study may be subject to potential selection bias due to recruiting from a single tertiary referral center, which could impact the clinical relevance. Further research is required to establish the cost-effectiveness and to provide more detailed guidance on clinical workflows.

In conclusion, this study demonstrates that POC measurement of adalimumab concentrations and anti-drug antibodies using the lateral-flow cartridge exhibits clinical significance and reliability comparable to those of existing commercially available ELISA kits. The results correlated well with the disease activity and clinical characteristics of patients, suggesting potential clinical relevance. Nonetheless, conducting larger, multicenter studies is essential to validate the observed associations and confirm the device’s applicability across diverse patient populations.

## 4. Materials and Methods

### 4.1. Study Design and Population

Ninety-six patients receiving adalimumab injections and attending the Department of Rheumatology at Severance Hospital, a tertiary hospital in Seoul, South Korea, were consecutively recruited based on their order of visits between 27 August 2023 and 14 October 2023. The clinical characteristics of the patients were retrieved from the medical records, and baseline data for ANA and RF were retrospectively obtained from the pre-adalimumab treatment records. Disease activity was assessed using the standard disease activity scale for each diagnosis and categorized arbitrarily by the investigator as none (remission), mild, moderate, or severe. One year following the commencement of enrollment, we assessed the current status of ADA continuation within the study populations.

### 4.2. Sample Collection and Measurements

Serum samples were collected on the day of the visit to measure anti-drug antibodies and drug concentrations, including inflammation levels. Three commercially available ELISA methods (RIDASCREEN^®^ ADM Monitoring [R-Biopharm AG, Germany], IDKmonitor^®^ Adalimumab Drug Level [Immundiagnostik AG, Germany], and LISA-TRACKER Duo Adalimumab [Theradiag, France]) were used to measure adalimumab concentration, and two ELISA kits (IDKmonitor^®^ Adalimumab free ADA [Immundiagnostik AG, Germany] and LISA-TRACKER Duo Adalimumab [Theradiag, France]) were used for AAA detection. ELISA was performed according to the manufacturer’s protocols, ensuring consistency in the assay procedures. The results were compared with the measurements obtained using the commercially available AFIAS Adalimumab and AFIAS Free Anti-Adalimumab (Boditech Med Inc., Chuncheon, Republic of Korea).

### 4.3. AFIAS POC Assays and Reagents

The AFIAS assays (AFIAS Adalimumab and AFIAS Free Anti-Adalimumab) were conducted using the AFIAS-10 analyzer (Boditech Med Inc., Chuncheon, Republic of Korea), an automated, integrated system designed to efficiently process samples from input to result output, ensuring ease of use and high-throughput capacity. The platform enables the simultaneous execution of up to 10 tests without manual intervention. For each test, 100 µL of the sample was loaded into the sample well, and the system automatically processed the assay, including incubation, detection, and interpretation of the results. The operation and basic principles of the AFIAS are based on fluorescence-based lateral flow immunoassay. AFIAS Adalimumab assay results were obtained after 10 min, while AFIAS Free Anti-Adalimumab results were available after 12 min. The measurement ranges for both the AFIAS and ELISA are summarized in Supplementary Tables S8 and S9.

The assays utilize disposable all-in-one cartridges containing fluorescence-labeled antibodies, buffers, and test strips, which enable fully automated sample-to-result processing without manual reagent preparation. Quality control materials supplied by the manufacturer were used according to protocol. Additionally, the World Health Organization (WHO) international standard material for Adalimumab (NIBSC code 17/236) was employed as a reference to validate the assay.

### 4.4. Analytical Validation

Analytical validation of the AFIAS Adalimumab assay was performed according to CLSI guidelines. Precision assessment, including repeatability, within-laboratory precision, and lot-to-lot reproducibility, was evaluated at four concentrations (2, 5, 8, and 20 µg/mL) using three reagent lots. Replicate measurements were conducted to calculate coefficients of variation (CV) and recovery rates based on the WHO international standard (Supplementary Tables S1 and S2). To assess inter-operator variability and minimize potential bias, two trained laboratory technicians independently performed all AFIAS and ELISA measurements under standardized protocols. Inter-operator precision was analyzed by comparing replicate results (Supplementary Table S1).

### 4.5. Statistical Analysis

For baseline and proportional bias between the measurement methods, both the Passing–Bablok analysis and Bland–Altman plots were used. Passing–Bablok regression was used to determine the intercept and slope of the linear regression using the equation y = a + bx [28]. To assess the agreement between the two methods in quantification, the Bland–Altman analysis was performed by examining the mean difference and percent mean difference, establishing limits of agreement (LOAs) [29]. LOAs were calculated as the mean difference ± 1.96 times the standard deviation of the differences. The agreement was sufficient if the concentration difference was within the LOA for 67% of the sample pairs [29]. Lin’s concordance correlation coefficient (ρ_c_) was used to measure agreement or reliability between two sets of continuous measurements. The utility of ρ_c_ lies in its ability to capture both the precision (Pearson’s correlation coefficient) and accuracy (Cβ) of the agreement between the two sets of agreements. Cβ is a bias that considers both the systematic difference (bias) and dispersion (variability) between measurements. Altman considered the interpretation of ρ_c_ > 0.8 to be excellent, while McBride indicated that <0.9 suggests poor agreement [30]. Intraclass correlation coefficients (ICCs) were calculated for the adalimumab concentration values using a two-way mixed-effects model to investigate the consistency of the results between our method and commercially available ELISA kits. ICC is a measure of reliability and ranges from 0 to 1. Generally, it describes the ratio of variance of interest (i.e., due to patient differences) to the total variance (i.e., variance of interest + noise). For example, if the unwanted variance (noise) is equal to or larger than the variance due to patient differences, the reliability is considered poor, and the ICC will be ≤0.5. In contrast, ICC values above 0.8 or 0.9 are often regarded as indicators of good or excellent reliability [30,31]. To examine the dichotomous determination of AAA, the percent agreement was calculated. Cohen’s or Fleiss’s kappa statistics were calculated for decision agreements between two or more than three groups to detect AAAs [28]. The kappa value was interpreted according to the criteria proposed by Landis et al. [32]: k < 0, no agreement; k (0.00–0.20), slight agreement; k (0.21–0.40), fair agreement; k (0.41–0.60), moderate agreement; k (0.61–0.80), substantial agreement; and k (0.81–1.00): almost perfect agreement.

Regarding the necessary sample count for calculating ICC, a sample size of 53 participants with two observations per participant achieved 80% power to detect an ICC of 0.5 under the alternative hypothesis when the intraclass correlation under the null hypothesis was 0.2, using an F-test with a significance level of 0.05. For kappa, in a test for agreement between two raters using the kappa statistic, a sample size of 53 participants achieved 80% power to detect a true kappa value of 0.7 in a test of H0: kappa = κ0 vs. H1: kappa ≠ κ0 when there were three categories with frequencies equal to 0.2, 0.3, and 0.5. The power calculation was based on a significance level of *p* < 0.05.

Regarding clinical relevance, we analyzed the associations of drug concentrations and anti-drug antibody detection with clinical characteristics. The chi-square test or Fisher’s exact test was performed as appropriate to compare categorical variables. For normally distributed continuous variables, a *t*-test was used; otherwise, the rank-sum test or Wilcoxon signed-rank test was used for paired samples. Linear regression analysis was performed to calculate the coefficient values for each variable of adalimumab concentration, and logistic regression analysis was performed for the development of anti-drug antibodies or disease activity. Backward selection was employed to identify the pertinent variables for the development of AAA in the context of multivariate logistic analysis. In addition, to assess the longitudinal outcomes associated with the retention of ADA within the study group, we performed both univariate and multivariate Cox regression analyses. The proportional-hazard assumption was verified using Schoenfeld residuals. By employing initial ADA concentrations and AAA detection results, we evaluated the survival curve for the continuation of ADA administration. Moreover, we calculated the estimated hazard function plots over the observed failure times, categorizing the patient group based on ADA concentration and the presence of AAA. Statistical significance was set at *p* < 0.05.

All statistical analyses were performed using STATA for Windows version 18.0 (Stata Corp., College Station, TX, USA).

### 4.6. Ethics

The Institutional Review Board (IRB) of Severance Hospital approved this study (IRB: 2022-1531-001). All the patients provided informed consent for participation in the study. The study was conducted in full conformity with the current revision of the Declaration of Helsinki (2013) and in accordance with local guidelines and regulations.

## Figures and Tables

**Table 1:** Precision of AFIAS adalimumab.

Expected Conc.	Repeatability			Within-Laboratory Precision			Lot to Lot Precision		
[μg/mL]	Mean [μg/mL]	SD	CV (%)	Mean [μg/mL]	SD	CV (%)	Mean [μg/mL]	SD	CV (%)
2	2.01	0.15	7.4	1.98	0.14	6.9	1.99	0.13	6.4
5	5.06	0.35	6.9	4.99	0.31	6.3	5	0.32	6.5
8	8.19	0.64	7.8	8.02	0.57	7.1	8.02	0.53	6.6
20	19.84	1.35	6.8	19.69	1.28	6.5	19.8	1.27	6.4

CV—coefficient value; SD—standard deviation.

**Table 2:** Accuracy of AFIAS adalimumab.

Sample No.	Expected Value [μg/mL]	Lot 1	Lot 2	Lot 3	Mean [μg/mL]	Recovery (%)
1	45.05	43.13	45.31	43.27	43.9	97%
2	35.05	32.77	32.51	32.88	32.72	93%
3	25.05	24.02	23.3	23.72	23.68	95%
4	15.05	14.37	14.2	14.01	14.19	94%
5	7.55	7.26	7.34	7.29	7.3	97%
6	1.05	1	0.96	1	0.99	94%

**Table 3:** Demographic characteristics of the patients.

	Diagnosis			
	Rheumatoid Arthritis	Ankylosing Spondylitis	Total	*p*-Value
N	58 (60.4%)	38 (39.6%)	96 (100.0%)	
Age (Years)	60.0 (11.0)	41.8 (13.1)	52.8 (14.8)	<0.001
Sex				
Men	11 (19.0%)	31 (81.6%)	42 (43.8%)	<0.001
Women	47 (81.0%)	7 (18.4%)	54 (56.2%)	
BMI, kg/m^2^	23.6 (3.9)	25.1 (3.4)	24.2 (3.8)	0.075
Adalimumab duration ^a^, year	4.6 (4.2)	5.5 (4.6)	5.0 (4.4)	0.312
Injection interval, weeks ^b^	2.1 (0.2)	2.5 (1.5)	2.2 (1.0)	0.038
ANA				
Negative	22 (43.1%)	30 (88.2%)	52 (61.2%)	<0.001
Positive	29 (56.9%)	4 (11.8%)	33 (38.8%)	
RF				
Negative	14 (24.1%)	35 (94.6%)	49 (52.1%)	<0.001
Positive	44 (75.9%)	2 (5.4%)	45 (47.9%)	
RF value, IU/mL	137.5 (226.7)	5.8 (13.9)	85.6 (187.6)	<0.001
Anti-CCP_Ab				
Negative	11 (19.3%)	33 (100.0%)	44 (48.9%)	<0.001
Positive	46 (80.7%)	0 (0.0%)	46 (51.1%)	
Anti-CCP-Ab value, U/mL	297.1 (477.4)	0.0 (0.0)	181.7 (399.5)	<0.001
MTX				
No	22 (37.9%)	33 (86.8%)	55 (57.3%)	<0.001
Yes	36 (62.1%)	5 (13.2%)	41 (42.7%)	
Steroid				
No	33 (56.9%)	36 (94.7%)	69 (71.9%)	<0.001
Yes	25 (43.1%)	2 (5.3%)	27 (28.1%)	
CRP, mg/dL	0.4 (1.3)	0.1 (0.3)	0.3 (1.0)	0.154
ESR, mm/h	31.1 (30.1)	8.2 (8.9)	22.0 (26.5)	<0.001
Disease activity				
No	33 (56.9%)	29 (76.3%)	62 (64.6%)	0.139
Mild	20 (34.5%)	9 (23.7%)	29 (30.2%)	
Moderate	3 (5.2%)	0 (0.0%)	3 (3.1%)	
Severe	2 (3.4%)	0 (0.0%)	2 (2.1%)	

^a^ Duration—years from the first adalimumab injection to the day of enrollment; ^b^ interval—interval between adalimumab injections. ANA—antinuclear antibody; anti-CCP Ab—anti-cyclic citrullinated peptide antibody; BMI—body mass index; CRP—C-reactive protein; ESR—erythrocyte sedimentation rate; MTX—methotrexate; RF—rheumatoid factor.

**Table 4:** Comparison of adalimumab concentrations among measurement kits.

	Bland–Altman		Passing–Bablok (Y = A + B × X)			
	Difference	95%CI	A	95%CI	B	95%CI
AFIAS-RIDA	0.8513 ± 1.4171	0.551 to 1.1515	0.2498	−0.1539 to 0.6407	1.0462	0.9822 to 1.1102
AFIAS-IDK	2.0343 ± 2.5939	1.4971 to 2.5715	−1.3432	−1.7357 to −0.868	1.391	1.324 to 1.458
AFIAS-LISA	2.7589 ± 2.496	2.2331 to 3.2847	−0.4761	−0.1467 to 0.9243	1.2735	1.1893 to 1.3704

AFIAS—AFIAS Adalimumab; CI—confidence interval; IDK—IDKmonitor^®^ Adalimumab Drug Level; LISA—LISA TRACKER Duo; RIDA—RIDASCREEN^®^ ADM Monitoring. In this Passing-Bablok regression function, Y means the concentration measured by AFIAS and X means the concentration measured by the other ELISA kit (RIDA, IDK, or LISA).

**Table 5:** Comparison of ICC and kappa values between ELISA kits and point-of-care AFIAS.

ADL Concentration				AAA Detection			
**Test Kit**	**ICC Value ^a^**	**95%CI**	**Interpretation**	**Test Kit**	**Kappa**	**95%CI**	**Interpretation**
RIDA ^b^ vs. AFIAS ^c^	0.9699	(0.955–0.98)	Excellent	AFIAS ^f^ vs. IDK ^g^	0.810 ^h^	(0.629–0.99)	Almost perfect
IDK ^d^ vs. AFIAS ^c^	0.8868	(0.834–0.924)	Good	AFIAS ^f^ vs. LISA ^e^	0.753 ^h^	(0.546–0.96)	Substantial agreement
LISA ^e^ vs. AFIAS ^c^	0.8954	(0.845–0.93)	Good	LISA ^e^ vs. IDK ^g^	0.77 ^h^	(0.577–0.963)	Substantial agreement
All (RIDA ^b^, IDK ^d^, LISA ^e^, AFIAS ^c^)	0.9026	(0.868–0.931)	Good	ALL (AFIAS ^f^, IDK ^g^, LISA ^e^)	0.778 ^i^	(0.702–0.932)	Substantial agreement

^a^ Two-way mixed-effects model; ^b^ RIDASCREEN^®^ ADM monitoring; ^c^ AFIAS Adalimumab; ^d^ IDKmonitor^®^ Adalimumab Drug Level; ^e^ LISA-TRACKER Duo Adalimumab; ^f^ AFIAS Free Anti-Adalimumab; ^g^ IDKmonitor^®^ Adalimumab-free ADA; consistency of measurements; ^h^ Cohen’s Kappa; ^i^ Fleiss’ Kappa. AAA—anti-adalimumab antibody; ADL—adalimumab; ICC—intraclass correlation coefficient.

**Table 6:** Differences in clinical characteristics according to the presence of anti-adalimumab antibodies.

	Anti-Adalimumab Antibodies			
	Negative	Positive	Total	*p*-Value
N	85 (88.5%)	11 (11.5%)	96 (100%)	
Age	52.6 (14.9)	54.6 (15.0)	52.8 (14.8)	0.665
Sex				
Men	39 (45.9%)	3 (27.3%)	42 (43.8%)	0.242
Women	46 (54.1%)	8 (72.7%)	54 (56.2%)	
BMI	24.1 (3.7)	25.1 (4.3)	24.2 (3.8)	0.396
Diagnosis				
AS	35 (41.2%)	3 (27.3%)	38 (39.6%)	0.375
RA	50 (58.8%)	8 (72.7%)	58 (60.4%)	
Duration ^a^	5.0 (4.4)	4.9 (3.9)	5.0 (4.4)	0.942
Interval ^b^	2.2 (1.0)	2.1 (0.3)	2.2 (1.0)	0.647
ANA				
Negative	49 (65.3%)	3 (30.0%)	52 (61.2%)	0.031
Positive	26 (34.7%)	7 (70.0%)	33 (38.8%)	
Rheumatoid factor				
Negative	45 (54.2%)	4 (36.4%)	49 (52.1%)	0.265
Positive	38 (45.8%)	7 (63.6%)	45 (47.9%)	
Rheumatoid factor, IU/mL	71.6 (175.2)	191.7 (248.1)	85.6 (187.6)	0.045
Anti-CCP				
Negative	41 (51.9%)	3 (27.3%)	44 (48.9%)	0.126
Positive	38 (48.1%)	8 (72.7%)	46 (51.1%)	
Anti-CCP Antibody, U/ml	163.2 (372.7)	338.7 (585.1)	181.7 (399.5)	0.215
CRP, mg/L	0.3 (1.0)	0.6 (0.8)	0.3 (1.0)	0.391
ESR, mm/h	20.0 (25.6)	37.5 (29.5)	22.0 (26.5)	0.038
MTX				
No	46 (54.1%)	9 (81.8%)	55 (57.3%)	0.081
Yes	39 (45.9%)	2 (18.2%)	41 (42.7%)	
Steroid				
No	62 (72.9%)	7 (63.6%)	69 (71.9%)	0.518
Yes	23 (27.1%)	4 (36.4%)	27 (28.1%)	
Disease activity				
No	58 (68.2%)	4 (36.4%)	62 (64.6%)	0.003
Mild	25 (29.4%)	4 (36.4%)	29 (30.2%)	
Moderate	1 (1.2%)	2 (18.2%)	3 (3.1%)	
Severe	1 (1.2%)	1 (9.1%)	2 (2.1%)	

^a^ duration—years from the first adalimumab injection to the day of enrollment; ^b^ interval—interval between adalimumab injections. ANA—antinuclear antibody; BMI—body mass index; CCP—cyclic citrullinated peptide; CRP—C-reactive protein; ESR—erythrocyte sedimentation rate; MTX—methotrexate; RA—rheumatoid arthritis.

## Data Availability

The original contributions presented in this study are included in the article and Supplementary Material. Further inquiries can be directed to the corresponding author(s).

## Supplementary Materials

The following supporting information can be downloaded at https://www.mdpi.com/article/10.3390/ijms26178741/s1.

## Abbreviations

The following abbreviations are used in this manuscript:

POC	Point of Care.
AFIAS	AFIAS Adalimumab.
ELISA	Enzyme-Linked Immunosorbent Assay.
AAA	Anti-Adalimumab Antibodies.
RA	Rheumatoid Arthritis.
AS	Ankylosing Spondylitis.
TNF	Tumor Necrosis Factor.
CLSI	Clinical & Laboratory Standard Institute.
BMI	Body Mass Index.
ANA	Antinuclear Antibody.
RF	Rheumatoid Factor.
ADL	Adalimumab.
ICC	Intraclass Correlation Coefficient.
CI	Confidence Interval.
CCP	Cyclic Citrullinated Peptide.
CRP	C-Reactive Protein.
ESR	Erythrocyte Sedimentation Rate.
MTX	Methotrexate.
PD	Prednisolone.
