# Supplementary material for: Clinical Implications of Point-of-Care Measurement of Adalimumab Concentration and Anti-Adalimumab Antibodies in Patients with Rheumatoid Arthritis and Ankylosing Spondylitis
Source: Int J Mol Sci. 2025 Sep 8;26(17):8741. doi: 10.3390/ijms26178741 (PMC12428896; doi:10.3390/ijms26178741)
Supplement: Supplementary file 1 [file ijms-26-08741-s001.zip › ijms-3813424-supplementary.pdf]

Supplementary Materials

Table of Contents

SUPPLEMENTARY TABLE S1. INTER-OPERATOR PRECISION OF AFIAS ADALIMUMAB 2
SUPPLEMENTARY TABLE S2. RESULTS OF ADALIMUMAB CONCENTRATION AND DETECTION OF ANTI-ADALIMUMAB ANTIBODIES 3
SUPPLEMENTARY TABLE S3. AGREEMENT OF ADL CONCENTRATION CLASSIFIED BY THERAPEUTIC RANGE 4
SUPPLEMENTARY TABLE S4. RESULTS OF ADALIMUMAB CONCENTRATION AND ANTI-ADALIMUMAB ANTIBODY DETECTION BY DISEASE 5
SUPPLEMENTARY TABLE S5. ODDS RATIO FOR ANTI-DRUG ANTIBODY FOR EACH KIT 6
SUPPLEMENT TABLE S6. CLINICAL CHARACTERISTICS BY DRUG DISCONTINUATION DURING FOLLOW-UP. 7
SUPPLEMENTARY TABLE S7. RESULTS OF COX REGRESSION ANALYSIS FOR DRUG RETENTION. 9
SUPPLEMENTARY TABLE S8. CHARACTERISTICS OF ASSAYS USED FOR METHOD COMPARISON 10
SUPPLEMENTARY TABLE S9. ANALYTICAL SPECIFICATION OF AFIAS 11
SUPPLEMENTARY FIGURE S1. MOUNTAIN PLOT FOR THE FREQUENCY OF DIFFERENCE BETWEEN KITS 12
SUPPLEMENTARY FIGURE S2. REGRESSION COEFFICIENT FOR ADALIMUMAB DRUG CONCENTRATION (UNIVARIATE) 13
SUPPLEMENTARY FIGURE S3. ODDS RATIO FOR DISEASE ACTIVITY 14
SUPPLEMENTARY FIGURE S4. KAPLAN-MEIER SURVIVAL ESTIMATES FOR ADA RETENTION. 16

Supplementary Table S1. Inter-operator Precision of AFIAS Adalimumab

**Expected Conc. [μg/mL]**	**Operator 1 Mean**	**Operator 2 Mean**	**Inter-operator SD**	**CV (%)**
2	2.16	2.17	0.19	8.8
5	5.39	5.27	0.49	9.2
8	8.98	8.65	0.70	7.9
20	22.03	20.12	1.52	7.2

Supplementary Table S2. Results of adalimumab concentration and detection of anti-adalimumab antibodies

Adalimumab concentration	RIDA	IDK	LISA	AFIAS	TOTAL
Mean ± SD	9.966±5.524	8.023±4.683	7.944±4.744	10.377±6.246	9.066±5.426
Median (min–max)	9.331 (0.564 to 28.368)	7.399 (0.596 to 22.927)	7.405 (0.420 to 19.361)	9.375 (0.340 to 30.990)	8.370 (0.34 to 30.99)
Anti-adalimumab antibodies					
Negative	NA	83	84	85	NA
Positive	NA	13	12	11	NA

AFIAS, AFIAS Adalimumab; IDK, IDKmonitor® Adalimumab Drug Level; LISA, LISA-TRACKER Duo Adalimumab; RIDA, RIDASCREEN® ADM Monitoring

Supplementary Table S3. Agreement of ADL concentration classified by therapeutic range

		RIDASCREEN		
	n	Subtherapeutic	Therapeutic	Supratherapeutic
AFIAS	Subtherapeutic	23	1	0
	Therapeutic	3	35	1
	Supratherapeutic	0	4	29
			Agreement: 90.6%	Kappa: 0.8570

		IDKmonitor		
	n	Subtherapeutic	Therapeutic	Supratherapeutic
AFIAS	Subtherapeutic	24	0	0
	Therapeutic	4	35	0
	Supratherapeutic	0	14	19
			Agreement: 81.3%	Kappa: 0.7123

		LISA		
	n	Subtherapeutic	Therapeutic	Supratherapeutic
AFIAS	Subtherapeutic	24	0	0
	Therapeutic	10	29	0
	Supratherapeutic	1	14	18
			Agreement: 74%	Kappa: 0.6069

AFIAS, AFIAS Adalimumab; IDK, IDKmonitor® Adalimumab Drug Level; LISA, LISA-TRACKER Duo Adalimumab; RIDA, RIDASCREEN® ADM Monitoring

Supplementary Table S4. Results of adalimumab concentration and anti-adalimumab antibody detection by disease

	Diagnosis				Total		
	RA		AS				p-value
N (%)		58 (60.42%)		38 (39.58%)		96 (100%)	
Anti-drug antibody							
AFIAS							
Negative		50 (86.21%)		35 (92.11%)		85 (88.54%)	0.37
Positive		8 (13.79%)		3 (7.89%)		11 (11.46%)	
IDK							
Negative		47 (81.03%)		36 (94.74%)		83 (86.46%)	0.06
Positive		11 (18.97%)		2 (5.26%)		13 (13.54%)	
LISA							
Negative		48 (82.76%)		36 (94.74%)		84 (87.50%)	0.08
Positive		10 (17.24%)		2 (5.26%)		12 (12.50%)	
Drug concentration	Mean ± SD	Median (min–max)	Mean ± SD	Median (min–max)	Mean ± SD	Median (min–max)	
AFIAS	10.25±(6.44)	9.71 0.34-25.93	10.55±(6.04)	9.20 0.59-30.99	10.38±(6.25)	9.38 0.34-30.99	0.82
RIDA	9.79±(5.58)	9.58 0.56-22.33	10.21±(5.51)	9.14 0.63-28.37	9.97±(5.52)	9.33 0.56-28.37	0.72
IDK	7.48±(4.66)	7.26 0.60-20.55	8.85±(4.66)	8.17 1.11-22.93	8.02±(4.68)	7.40 0.60-22.93	0.16
LISA	8.13±(5.03)	7.48 0.45-19.36	7.70±(4.38)	6.97 0.42-19.32	7.94±(4.74)	7.40 0.42-19.36	0.68

RA, rheumatoid arthritis; AS, ankylosing spondylitis; AFIAS, AFIAS Adalimumab or AFIAS Free Anti-Adalimumab; IDK, IDKmonitor® Adalimumab Drug Level or IDKmonitor® Adalimumab Free ADA; LISA, LISA-TRACKER Duo Adalimumab; RIDA, RIDASCREEN® ADM Monitoring

Supplementary Table S5. Odds ratio for anti-drug antibody for each kit

AFIAS	Odds ratio (univariate)[a]	p-value	Odds ratio (multivariate)[b]	p-value
Age	1.010	0.661	0.961	0.273
Woman	2.261	0.251	5.170	0.164
duration	0.995	0.941	1.010	0.929
interval	0.763	0.666	1.095	0.889
RA (yes)	1.867	0.381	0.217	0.418
ANA (yes)	4.397	0.043	6.120	0.059
Log RF	1.398	0.045	1.602	0.106
MTX (yes)	0.262	0.099	0.123	0.052
PD (yes)	1.540	0.521	2.203	0.440
IDK				
Age	1.031	0.166	0.966	0.314
Woman	1.900	0.316	1.243	0.826
duration	0.981	0.789	1.023	0.818
interval	NA	NA	NA	NA
RA (yes)	4.213	0.072	0.918	0.962
ANA (yes)	6.125	0.011	5.195	0.065
Log RF	1.682	0.003	1.637	0.074
MTX (yes)	0.553	0.354	0.210	0.094
PD (yes)	2.531	0.129	2.186	0.369
LISA				
Age	1.031	0.178	1.003	0.923
Woman	4.545	0.060	11.720	0.048
duration	0.980	0.779	0.985	0.879
interval	NA	NA	NA	NA
RA (yes)	3.750	0.101	0.128	0.218
ANA (yes)	5.227	0.022	3.629	0.143
Log RF	1.541	0.011	1.556	0.100
MTX (yes)	0.952	0.938	0.861	0.860
PD (yes)	2.013	0.271	1.635	0.569

[a,b] Odds ratio from logistic regression for AAA

ANA, antinuclear antibody; interval, injection interval of adalimumab, weeks; duration, duration of adalimumab administration, years; MTX, methotrexate; PD, prednisolone; RA, rheumatoid arthritis; RF, rheumatoid factor

AFIAS, AFIAS Free Anti-Adalimumab; IDK, IDKmonitor® Adalimumab Free ADA; LISA, LISA-TRACKER Duo Adalimumab

Supplement Table S6. Clinical characteristics by drug discontinuation during follow-up.

	ADA Continued	ADA Discontinued	Total	p-value
N	88 (91.7%)	8 (8.3%)	96 (100 %)	
Follow-up time, days	290.5 ± 44.8	163.5 ± 115.5	279.9 ± 63.8	<0.001
Age	51.6 ± 14.7	65.9 ± 7.9	52.8 ± 14.8	0.008
Sex				
man	42 (47.7%)	0 (0 %)	42 (43.7%)	0.009
woman	46 (52.3%)	8 (100.000%)	54 (56.3%)	
bmi	24.3 ± 3.8	22.856 ± 3.5	24.189 ± 3.8	0.417
Diagnosis				
AS	38 (43.2%)	0 (0%)	38 (39.6%)	0.017
RA	50 (56.8%)	8 (100%)	58 (60.4%)	
ADA injection total duration	5.2 ± 4.4	2.2 ± 1.7	4.9 ± 4.4	0.058
Injection interval (weeks)	2.2 ± 1.0	2.0 ± 0.0	2.2 ± 1.0	0.511
ANA Positivity				
neg	49 (63.6%)	3 (37.5%)	52 (61.2%)	0.149
pos	28 (36.4%)	5 (62.5%)	33 (38.8%)	
RF positivity				
neg	47 (54.6%)	2 (25%)	49 (52.1%)	0.108
pos	39 (45.3%)	6 (75%)	45 (47.9%)	
Log(RF)	2.5 ± 2.0	4.2 ± 1.8	2.6 ± 2.0	0.018
CRP on the date of sample, mg/dL	0.2 ± 0.4	1.8 ± 3.1	0.3 ± 1.0	<0.001
ESR on the date of sample, mm/hr	17.6 ± 19.9	71 ± 40.1	22.0 ± 26.5	<0.001
Use of MTX				
no	51 (57.9%)	4 (50%)	55 (57.3%)	0.663
yes	37 (42.0%)	4 (50%)	41 (42.7%)	
Use of Steroid				
no	68 (77.3%)	1 (12.5%)	69 (71.9%)	<0.001
yes	20 (22.7%)	7 (87.5%)	27 (28.1%)	
Disease Activity				
no	60 (68.2%)	2 (25%)	62 (64.6%)	<0.001
mild	26 (29.5%)	3 (37.5%)	29 (30.2%)	
moderate	2 (2.3%)	1 (12.5%)	3 (3.1%)	
severe	0 (0%)	2 (25%)	2 (2.1%)	
Previous history of TNF use				
no	66 (75%)	6 (75%)	72 (75%)	1
yes	22 (25%)	2 (25%)	24 (25%)	
Group[a]				
Group 1	67 (76.1%)	3 (37.5%)	70 (72.9%)	0.085
Group 2	2 (2.3%)	0 (0.0%)	2 (2.1%)	
Group 3	12 (13.6%)	3 (37.0%)	15 (15.6%)	
Group 4	7 (7.9%)	2 (25.0%)	9 (9.4%)	

[a] Patients were categorized based on an ADA concentration threshold of 5 μg/mL and the presence of AAA into four groups: group 1 (high ADA, AAA-), group 2 (high ADA, AAA+), group 3 (low ADA, AAA-), and group 4 (low ADA, AAA+).
AAA, anti-adalimumab antibody; ADA, adalimumab; BMI, body mass index; AS, ankylosing spondylitis; RA, rheumatoid arthritis; ANA, antinuclear antibody; RF, rheumatoid factor; CRP, C-reactive protein; ESR, erythrocyte sedimentation rate; MTX, methotrexate; TNF, tumor necrosis factor inhibitor.

Supplementary Table S7. Results of Cox regression analysis for drug retention.

	Univariate Hazard Ratio	p-value	Multivariate Hazard Ratio	p-value
Age	1.070	0.015	1.139	0.029
Woman	>100	1.000	>100	.
RA	>100	1.000	>100	.
Duration[a]	0.759	0.095	0.723	0.027
Methotrexate	1.382	0.647	3.502	0.237
Group[b] 1	REF		REF	
Group 2	<0.1	0	>100	NA
Group 3	5.201	0.044	7.815	0.034
Group 4	5.627	0.059	7.755	0.046

[a] total duration of adalimumab administration (years). [b] Patients were categorized based on an ADA concentration threshold of 5 μg/mL and the presence of AAA into four groups: group 1 (high ADA, AAA-), group 2 (high ADA, AAA+), group 3 (low ADA, AAA-), and group 4 (low ADA, AAA+).

Notably, patients in group 3 and 4, characterized by low ADA concentrations, were most frequently associated with the discontinuation of ADA during follow-up. In multivariate analysis, older age and shorter duration of ADA injection period were appeared as risk factor for drug discontinuation.

AAA, anti-adalimumab antibody; ADA, adalimumab.

Supplementary Table S8. Characteristics of assays used for method comparison

Product	Company	Technique	Measurement range	
			ADL	AAA
IDKmonitor	Immunodiagnostik	ELISA	0.58–45 μg/mL	10–232 AU/mL
LISA TRACKER	Theradiag	ELISA	0.3–20 μg/mL	10–160 ng/mL
RIDASCREEN	R-biopharm	ELISA	0.5–48 μg/mL	2.5–1000 ng/mL
AFIAS	Boditech Med.	FLFIA	0.2–50 μg/mL	3–200 AU/mL

ELISA, enzyme-linked immunosorbent assay; FLFIA, fluorescence-based lateral flow immunoassay; ADL, adalimumab; AAA, anti-adalimumab antibody; AU, arbitrary unit

Supplementary Table S9. Analytical Specification of AFIAS

Parameter	AFIAS Adalimumab	AFIAS Free Anti-Adalimumab
Limit of blank (LoB)	0.06 μg/mL	1.5 AU/mL
Limit of detection (LoD)	0.09 μg/mL	3.0 AU/mL
Limit of quantification (LoQ)	0.20 μg/mL	-
Cut-off	-	10 AU/mL
Linear range	0.2–50 μg/mL	3–200 AU/mL
TAT	10 min	12 min
Sample type	Whole blood, Plasma, and serum	Whole blood, and serum
Anticoagulant	EDTA, heparin, and citrate	EDTA, heparin, and citrate

TAT, turn around time

Supplementary Figure S1. Mountain plot for the frequency of difference between kits

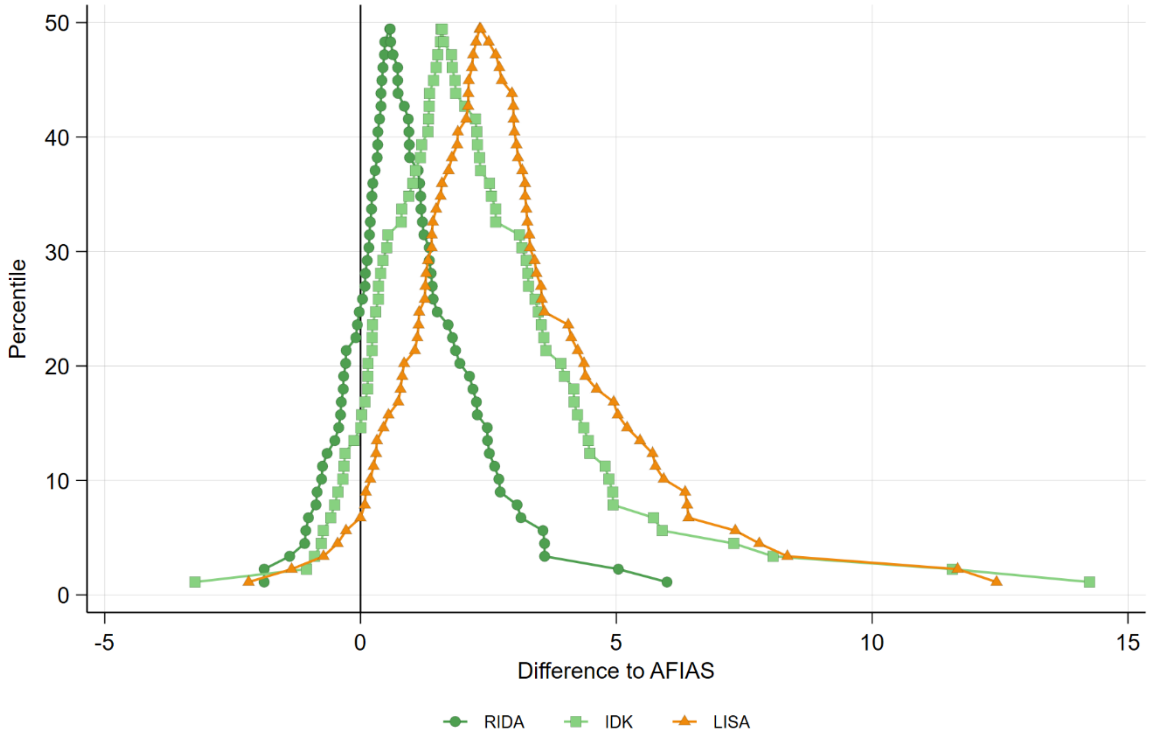

Differences in measurement of adalimumab concentration between AFIAS and each RIDA, IDK, or LISA were plotted with their values and the percentile. AFIAS-RIDA pairs showed a least difference. AFIAS, AFIAS Adalimumab; IDK, IDKmonitor® Adalimumab Drug Level; LISA, LISA-TRACKER Duo Adalimumab; RIDA, RIDASCREEN® ADM Monitoring

Supplementary Figure S2. Regression coefficient for adalimumab drug concentration (univariate)

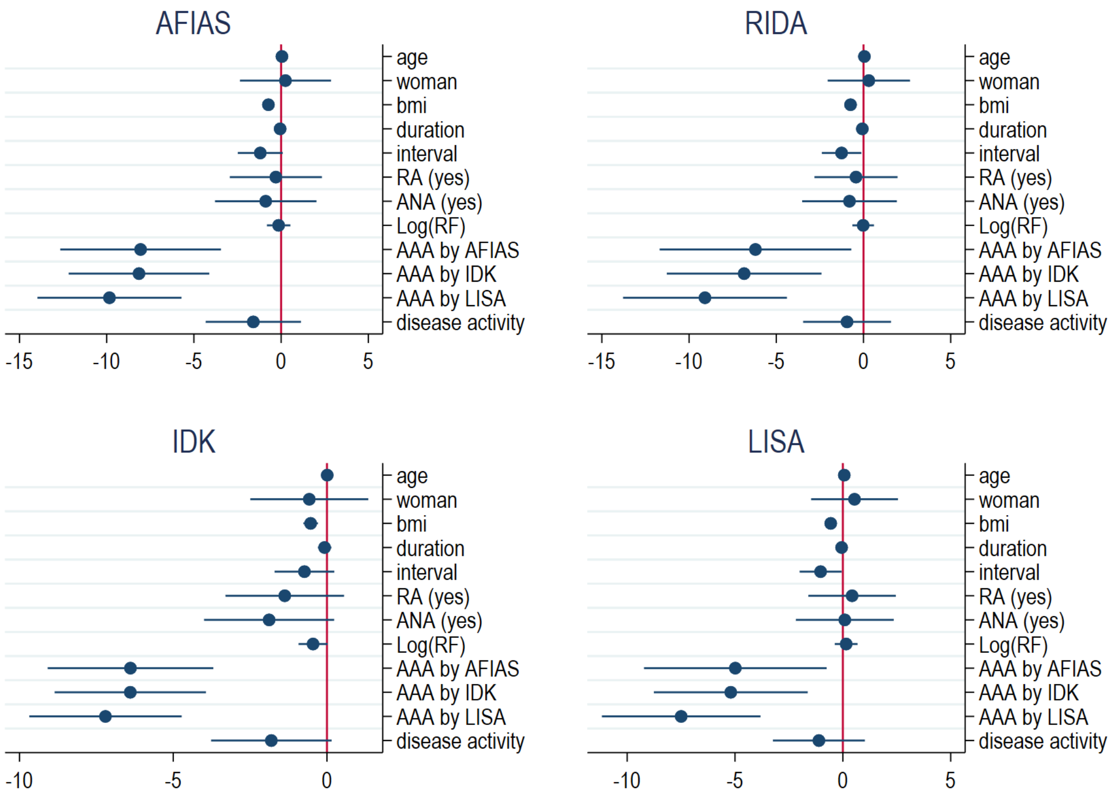

AAA, anti-drug antibody; bmi, body mass index; disease activity, moderate or severe disease activity; duration, duration of adalimumab administration; interval, injection interval of adalimumab, weeks; Log(RF), log function of rheumatoid factor quantification;

AFIAS, AFIAS Adalimumab or AFIAS Free Anti-Adalimumab; IDK, IDKmonitor® Adalimumab Drug Level or IDKmonitor® Adalimumab Free ADA; LISA, LISA-TRACKER Duo Adalimumab; RIDA, RIDASCREEN® ADM Monitoring

Supplementary Figure S3. Odds ratio for disease activity

A. Univariate

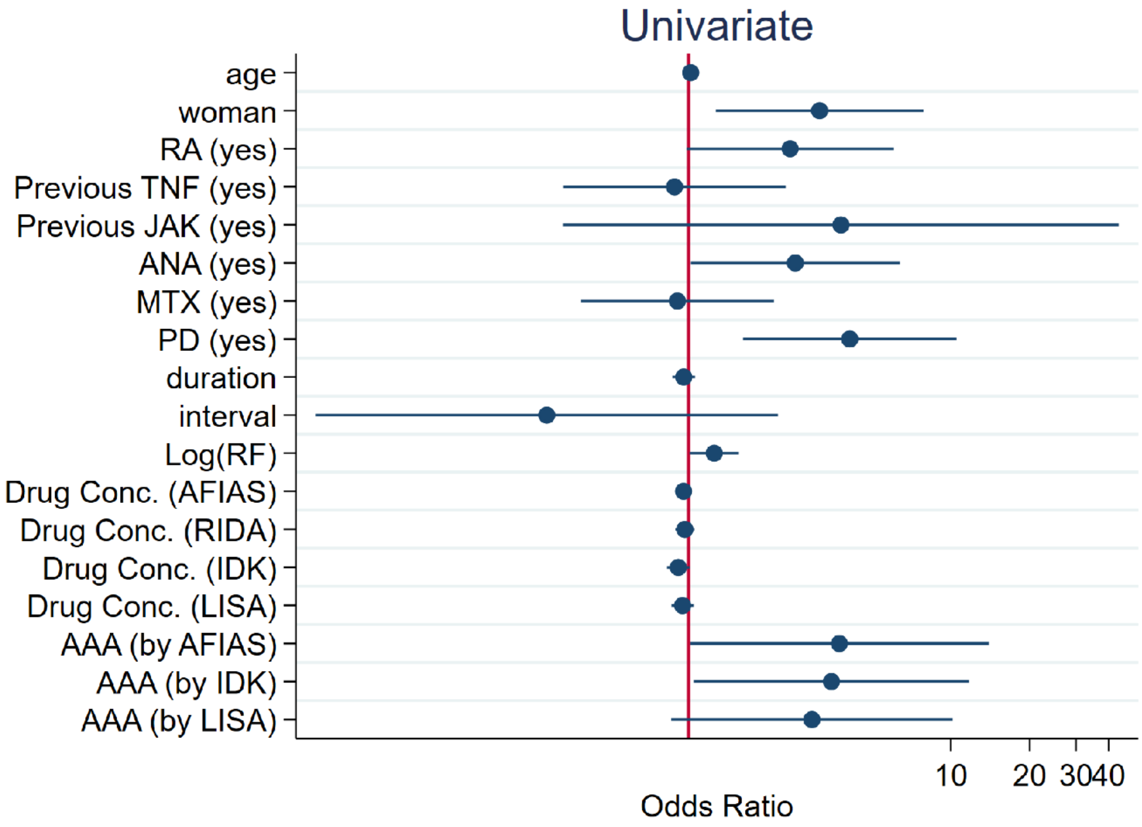

A

B. Multivariate

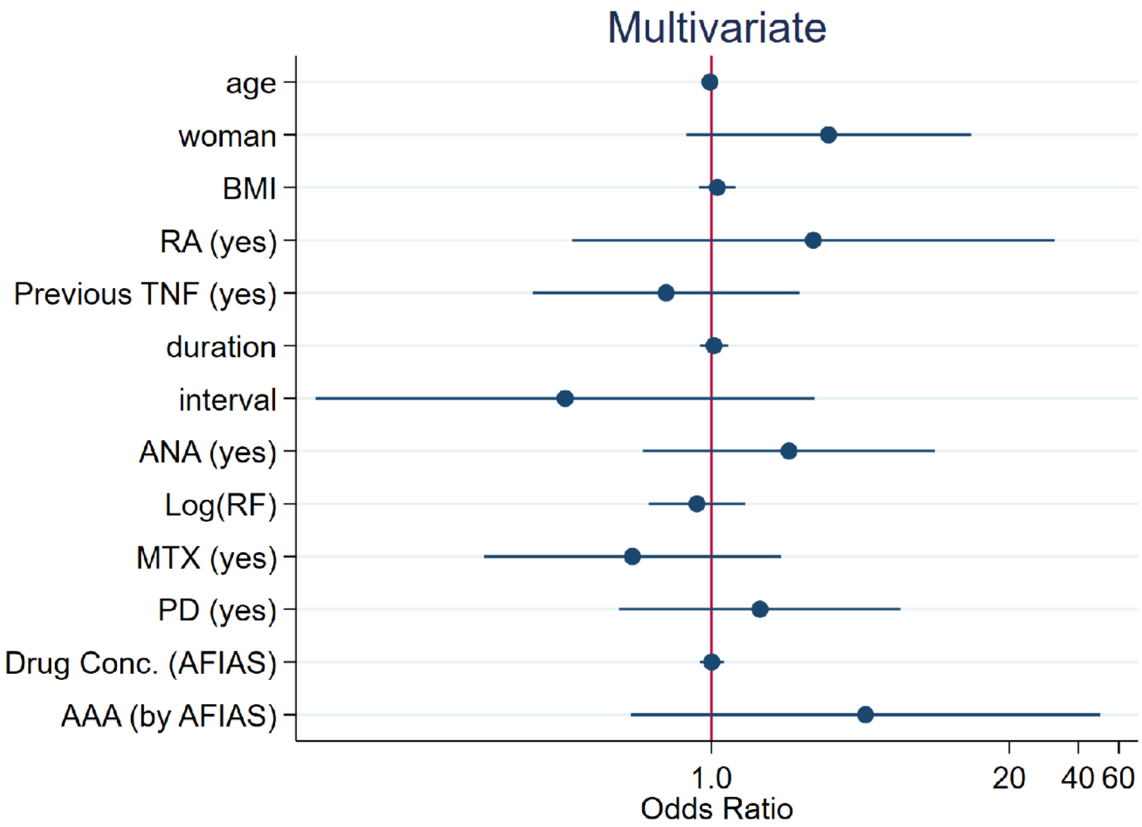

B

AAA, anti-drug antibody; Drug conc., adalimumab concentration; ANA, antinuclear antibody; BMI, body mass index; JAK, Janus kinase inhibitor; MTX, methotrexate; TNF, tumor necrosis factor inhibitor.
AFIAS, AFIAS Adalimumab or AFIAS Free Anti-Adalimumab; IDK, IDKmonitor® Adalimumab Drug Level or IDKmonitor® Adalimumab Free ADA; LISA, LISA-TRACKER Duo Adalimumab; RIDA, RIDASCREEN® ADM Monitoring. The x-axis is in log scale.

Supplementary Figure S4. Kaplan-Meier survival estimates for ADA retention.

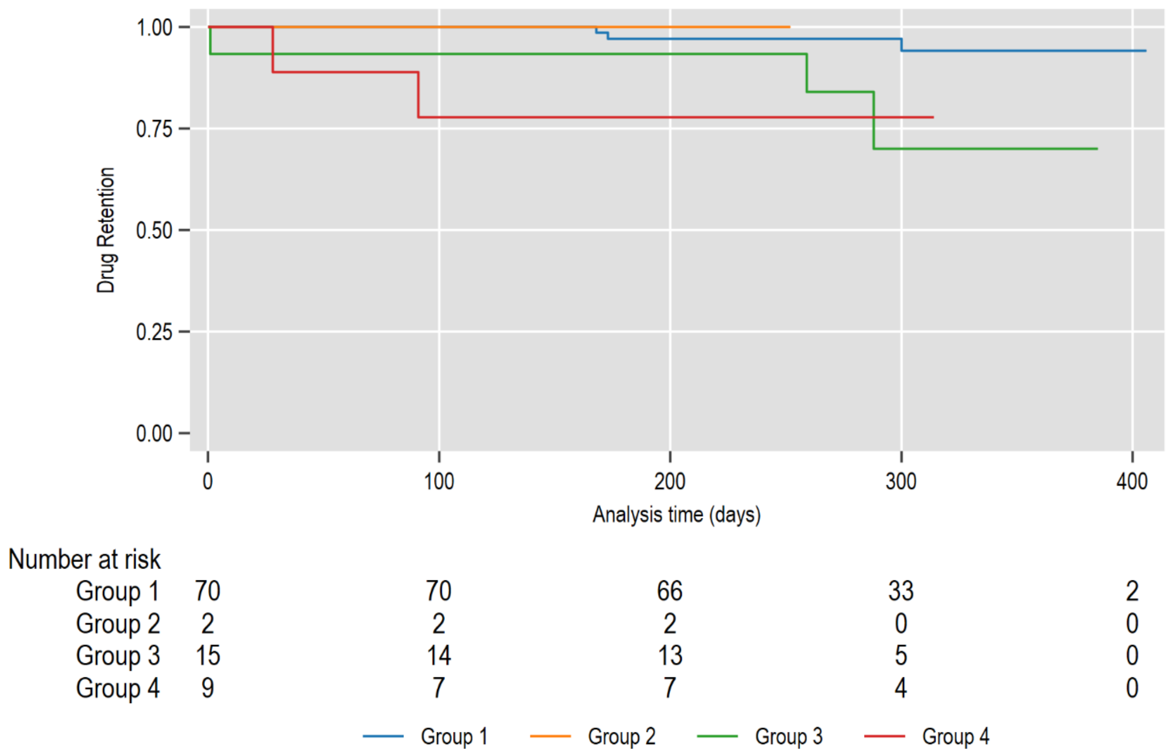

Patients were categorized based on an ADA concentration threshold of 5 μg/mL and the presence of AAA into four groups: group 1 (high ADA, AAA-), group 2 (high ADA, AAA+), group 3 (low ADA, AAA-), and group 4 (low ADA, AAA+).

AAA, anti-adalimumab antibody; ADA, adalimumab.
